# Systematic analysis of Baobaoqu fermentation starter for *Wuliangye* Baijiu by the combination of metagenomics and metabolomics

**DOI:** 10.3389/fmicb.2022.1062547

**Published:** 2022-12-01

**Authors:** Qingmei Zhang, Guocheng Du, Jian Chen, Jianghua Li, Zongwei Qiao, Jia Zheng, Dong Zhao, Xinrui Zhao

**Affiliations:** ^1^Key Laboratory of Industrial Biotechnology, Ministry of Education, School of Biotechnology, Jiangnan University, Wuxi, Jiangsu, China; ^2^Science Center for Future Foods, Jiangnan University, Wuxi, Jiangsu, China; ^3^Wuliangye Yibin Co., Ltd., Yibin, Sichuan, China; ^4^Key Laboratory of Carbohydrate Chemistry and Biotechnology, Ministry of Education, Jiangnan University, Wuxi, Jiangsu, China; ^5^Jiangsu Province Engineering Research Center of Food Synthetic Biotechnology, Jiangnan University, Wuxi, Jiangsu, China; ^6^Engineering Research Center of Ministry of Education on Food Synthetic Biotechnology, Jiangnan University, Wuxi, Jiangsu, China

**Keywords:** Baobaoqu, Nong-flavor Baijiu, metagenomics, metabolomics, biomarker, flavor

## Abstract

Baobaoqu (BBQ) is a traditional fermenting power, which is widely applied in Nong-flavor Baijiu brewing. There are two different types of BBQ (premium BBQ and normal BBQ) used in industrial manufacture, but the reasons for the significant differences between two kinds of BBQ have not been clearly illuminated. In this study, the combination of metagenomics and metabolomics was performed to compare the differences in the composition of microbial communities and the components of flavors between premium BBQ and normal BBQ. The results showed that the glycosidase-producing microorganisms are the biomarkers of premium BBQ, contributing a better ability of carbon source utilization than normal BBQ. In addition, several important flavors (ethyl hexanoate, phenylethanol, ethyl acetate) were rich in normal BBQ, which have a significant positive correlation with the biomarkers (*Lactobacillus* and *Pichia kudriavzevii*) of normal BBQ. It suggests that the microbial community has an advantage in utilizing raw materials in premium BBQ, while the community was inclined to form flavors in normal BBQ. The differences between two types of BBQ at the microbial and flavor level have theoretical and practical guiding significance in the application of premium and normal BBQ and in the further improvements of taste and quality of Baijiu.

## Introduction

Baijiu is one of the oldest distilled spirits in the world, which occupies an important position in the development of Chinese alcoholic beverages. In 2020, the annual sales of Baijiu reached $ 91.8 billion in China (data from the National Bureau of the Statistics of the People’s Republic of China). According to the characteristics of flavor, Baijiu can be divided into three main types: Nong-flavor (NF), Jiang-flavor (JF), and Qing-flavor (QF; Liu and Sun, 2018). Among three types, Nong-flavor Baijiu accounted for a half of sales market due to their outstanding fragrant flavor, soft mouthfeel, and persistent aftertaste (Liu et al., 2018). As the representative brand of NF Baijiu, *Wuliangye* Baijiu has a brewing history of more than 4,000 years and is one of the most popular alcoholic beverages in China. With the increasing nutrient, healthy and tasty demand of consumers, the manufacturer of *Wuliangye* also needs to continuously improve their brewing techniques to increase the quality of *Wuliangye* in recent years.

*Wuliangye* Baijiu is fermented by a traditional solid simultaneous saccharification technology dependent on the special fermentation starter (called *Daqu* in China) that provides abundant microorganisms, enzymes, and flavor substances (Xu et al., 2017). *Wuliangye Daqu* (also known as *Baobaoqu*, BBQ) is obtained from pure wheat through solid-state fermentation and is one of the most crucial factors affecting the flavor of Baijiu (Zheng et al., 2018). The manufacturing operation of BBQ is a process of directed screening and enriching functional microorganisms. However, due to many uncertain influence factors, two grades of BBQ with significant differences in fermentation performance are produced in the same production process of BBQ. According to the strict physical, biochemical and sensory indicators, BBQ can be divided into premium BBQ (PBQ, higher saccharification power, fewer cracks, smooth surface) and normal BBQ (NBQ). In practical production, two grades of BBQ are mixed according to a certain proportion for Baijiu brewing. At present, the reasons for the differences in the quality of PBQ and NBQ remain unknown and are required to be illuminated. Uncovering the functional microbes and characteristic metabolites in PBQ and BBQ will facilitate the comprehensive understanding of different roles between two grades of BBQ and lay the foundation for further determination of the optimal proportion between PBQ and NBQ used in the brewing process of *Wuliangye* Baijiu.

In recent years, with the rapid development of omics technology, a series of studies have been carried out on the composition and succession of microbial communities in *Daqu* (Nie et al., 2015; Huang et al., 2018; Park et al., 2019; Wang et al., 2020; Wu et al., 2020). Wang et al. (2015), Guan et al. (2021), and Pang et al. (2018) analyzed the microbial community and succession of Maotai-flavor *Daqu*, Luzhou-flavor *Daqu,* and Qing-flavor *Daqu* by amplicon sequencing technology. Jin et al. (2019) analyzed the microbial composition and flavor components of Maotai-flavored *Daqu* and found that Bacillales, Lactobacillales, and *Aspergillus* were positively correlated with the formation of flavor substances (pyrazines and esters) through the correlation network analysis. However, based on amplicon sequencing, the depth of research is limited to the analysis of microbial communities, and its correlation analysis with metabolome data is insufficient. As an important supplement to the detailed genetic information of microbial communities, the metagenomic technology not only can simultaneously obtain the composition of microbial communities but also can provide the full sequences of genes. Yang et al. used metagenomic technology to reveal a variety of aroma-producing microorganisms in the manufacturing process of medium-temperature *Daqu* (Yang et al., 2021). Gan et al. revealed that Maotai mature *Daqu* was mainly composed of 16 kinds of microorganisms and *Bacillus* is closely related to the saccharification power of *Daqu* (Gan et al., 2019). In addition, to make the functional research of microorganisms more reliable for *Daqu*, the application of multi-omics techniques is necessary. Compared with the amplicon sequencing technology, the combination of metagenomics and metabolomics can be used to systematically analyze the functional microorganisms, genes, and key enzymes for the formation of specific flavor substances in two grades of BBQ.

In this study, the differences in fermentation performance between two grades of BBQ were analyzed. The microbial community and related functional genes of PBQ and NBQ were studied based on metagenomic technology. The differential analysis of the composition of enzyme-producing microorganisms was carried out to compare the ability to utilize raw materials for the two grades BBQ. To explore the difference in the performance of aroma formation between PBQ and NBQ, the metabolomics was carried out to analyze the volatile flavor substances and to identify the correlation between core microbial community and characteristic flavors. These findings are helpful to understand the essential reasons for the differences between PBQ and NBQ and are beneficial to stabilize and improve the quality of *Wuliangye* Baijiu.

## Materials and methods

### Chemicals and reagents

Na_2_HPO_4_, KH_2_PO_4_, NaCl, and KCl were purchased from Sino Pharm Chemical Reagent Co. (Shanghai, China). DNeasy PowerSoil Pro Kit was purchased from QIAGEN Co. (Germany).

### Sample collection

The matured samples of PBQ and NBQ were obtained from a traditional BBQ-making garden of *Wuliangye* Yibin Co., Ltd. in Sichuan, China. The collected BBQ samples were crushed by a pulverizer (CS-2000Y, Cosiai, China), sealed in a sterile bag, and stored at −20°C.

### DNA extraction

5 g of BBQ sample, 15 ml of PBS buffer and 3 glass beads were added into a 50 ml centrifuge tube and shaken well for 5 min. Then centrifuge at 150 g for 5 min, and take the supernatant in the centrifuge tube. In the following, add 5 ml of PBS buffer to the pellet and repeat the washing twice, centrifuge at 150 g for 5 min, and collect the supernatant. Centrifuge the collected supernatant at 10, 000 g for 10 min, and collect the bacterial pellet. DNA was extracted using DNeasy PowerSoil Pro Kit following the manual. The extracted DNA was tested for quality and concentration by 1% agarose gel electrophoresis and spectrophotometry. The Samples with high quality were stored at −20°C for subsequent experiments.

### Metagenomics sequencing

DNA was sheared to 300 bp by the Covaris ultrasonic crusher. To prepare the sequencing library, the fragments were dealt with end repair, a tailing, and were ligated to the Illumina compatible adapters. The libraries of DNA sequencing were sequenced by Illumina Hiseq platform at Allwegene Company (Beijing). After the operation, image analysis, base calling, and error estimation were performed utilizing Illumina Analysis Pipeline Version 2.6.

### Data analysis

The quality control of raw data was performed by using Trimmomatic (Bolger et al., 2014), including the removal of adapter sequences and low-quality reads. Filter the reads if they were with the adapter sequence, the ratio of uncertain base greater than 1%, and the content of low-quality base (Q ≤ 20) greater than 50%. And filter out the reads whose length was still less than 150 bp after quality control.

High-quality sequences were compared with the NR database^1^ and categorized into different taxonomic groups by DIAMOND tool (Buchfink et al., 2015). MEGAHIT (Li et al., 2015) was used to assemble the sequencing data, and the contigs below 500 bp were filtered out. Contigs were annotated and the open reading frames (ORFs) were predicted by Prodigal software (Hyatt et al., 2012), and the set of non-redundant gene was constructed by CD-HIT (Li et al., 2001). Bowtie (Langmead et al., 2009) was applied to count the abundance information of genes in different samples. The function of genes were annotated by using the information in the database KEGG. The alpha-diversity indices (ACE and Shanno) were calculated by R4.0.0. One-way analysis of variance (ANOVA) with Tukey’s test was used to compare bacterial and fungal diversity of PBQ and NBQ. Principle Component Analysis (PCA) was performed by R4.0.0 to investigate the differences in the microbial and genetic composition between PBQ and NBQ. LEfSe (Linear discriminant analysis Effect Size) was performed to reveal the significantly different microorganisms in the two grades of BBQ. The microorganisms with the value of LDA (Linear Discriminant Analysis) higher than 3.0 were defined as differential microorganisms.

### Detection of the volatile flavors in BBQ

Volatile compounds in BBQ samples were analyzed by headspace solid-phase microextraction coupled with gas chromatography–mass spectrometry (HS-SPME-GC–MS). Briefly, BBQ samples (1 g) mixed with 10 μl 2-octanol (10 mg/l) as an internal standard. The volatile compounds were extracted using an SPME fiber (50,30 μm DVB/CAR/PDMS, Supelco Co., Bellefonte, PA, United States) at 60°C for 30 min. The analyses of GC/MS were performed on the Burker SCIONSQ-456-GC (Burker, United States). The separation of compounds was achieved using a DB-Wax column (30.0 m × 0.25 mm × 0.25 μm, Agilent, United States). Helium was used as a carrier gas at a constant flow rate of 0.8 ml/min. The oven temperature was kept at 40°C for 2 min, rose to 100°C at a rate of 6°C/min, then rose to 230°C at a rate of 10°C/min, and held for 6 min. The mass spectrometer was run in electron impact mode with the electron energy set at 70 eV and a scan range of 33–400 m/z. The temperature of the MS source and quadrupole was 200°C and 230°C, respectively. Each compound was identified using the NIST library and Wiley library. The compounds with positive and negative matching degrees greater than 800 were selected. According to the ratio of peak area between internal standard and flavor substances, the relative concentration of volatile flavors of BBQ were calculated. Orthogonal Partial Least Squares Discriminant Analysis (OPLS-DA) was performed using SIMCA-14.1 software (Umetricus, Sweden) to investigate the differences in metabolites between PBQ and NBQ. Metabolites with the VIP (Variable Importance for the Projection) value greater than 1.0 were defined as differential metabolites.

The Spearman correlation indices (ρ) and *p* values were calculated to reveal the relationship between potentially functional microorganisms (the relative abundance ranked top 30 and the LDA value was higher than 3.0) and differential metabolites (VIP value >1.0), and only the correlations that |ρ| > 0.6 and *p* < 0.05 were defined as significantly related and were visualized by Cytoscape.

### Data access

The metagenomic raw data has been deposited in the Genome Sequence Archive in Beijing Institute of Genomics (BIG) Data Center, Chinese Academy of Sciences, under accession number PRJCA008653 which is publicly accessible.^2^

## Results and discussion

### The composition of microbial communities in PBQ and NBQ

A total of 75.38 Gbp of clean data were obtained after the quality control and were assembled *de novo*. On average, the percentage of Q30 (bases with the qualitative value greater than or equal to 30 for clean data) was 93.82%; the percentage of G and C bases in clean data is 43.20. Clean reads were further optimized by being mapped against the complete genome of *Triticum aestivum L*. to preliminarily remove the host sequences (Supplementary Table S1). The maximum contig length was 338, 807 bp, and the minimum contig length was 500 bp. After gene prediction, the gene catalog contained approximately 494,911 non-redundant unigenes.

**Figure 1:**
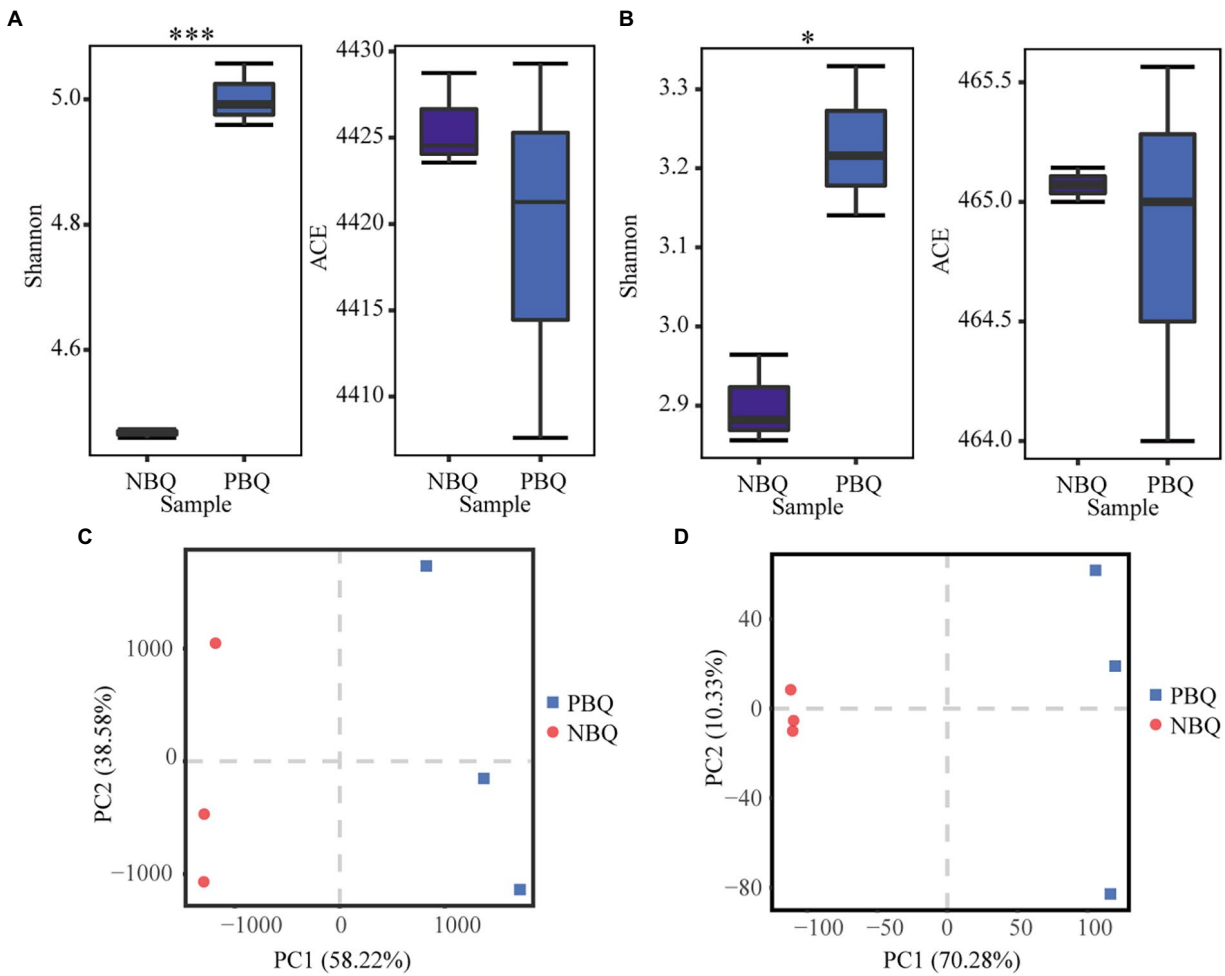
Alpha diversity analysis of bacterial community **(A)** and fungal community **(B)** of the two grades of BBQ, *p < 0.05, **p < 0.01, ***p < 0.001 determined by ANOVA; differences in the microbial **(C)** and genetic **(D)** composition of the two grades of BBQ based on PCA.

To compare the composition of microbial communities in the two grades of BBQ, the alpha-diversity indices (ACE and Shannon) were calculated to perform differential analysis on the species richness and diversity (Figures 1A,B). The results showed that the bacterial and fungal richness (ACE) of the two grades of BBQ was not significantly different, while the fungal and bacterial diversity (Shannon) of PBQ was significantly higher than NBQ. The differences in the microbial and genetic composition of PBQ and NBQ were compared based on PCA. The results showed that PBQ and NBQ were clustered separately on the PC1 axis, and the distance was far apart. It demonstrated that the species abundance and gene abundance of PBQ and NBQ differed significantly (Figures 1C,D). According to the above results, it suggests that there are significant differences in microbial composition and function between two grades of BBQ.

**Figure 2:**
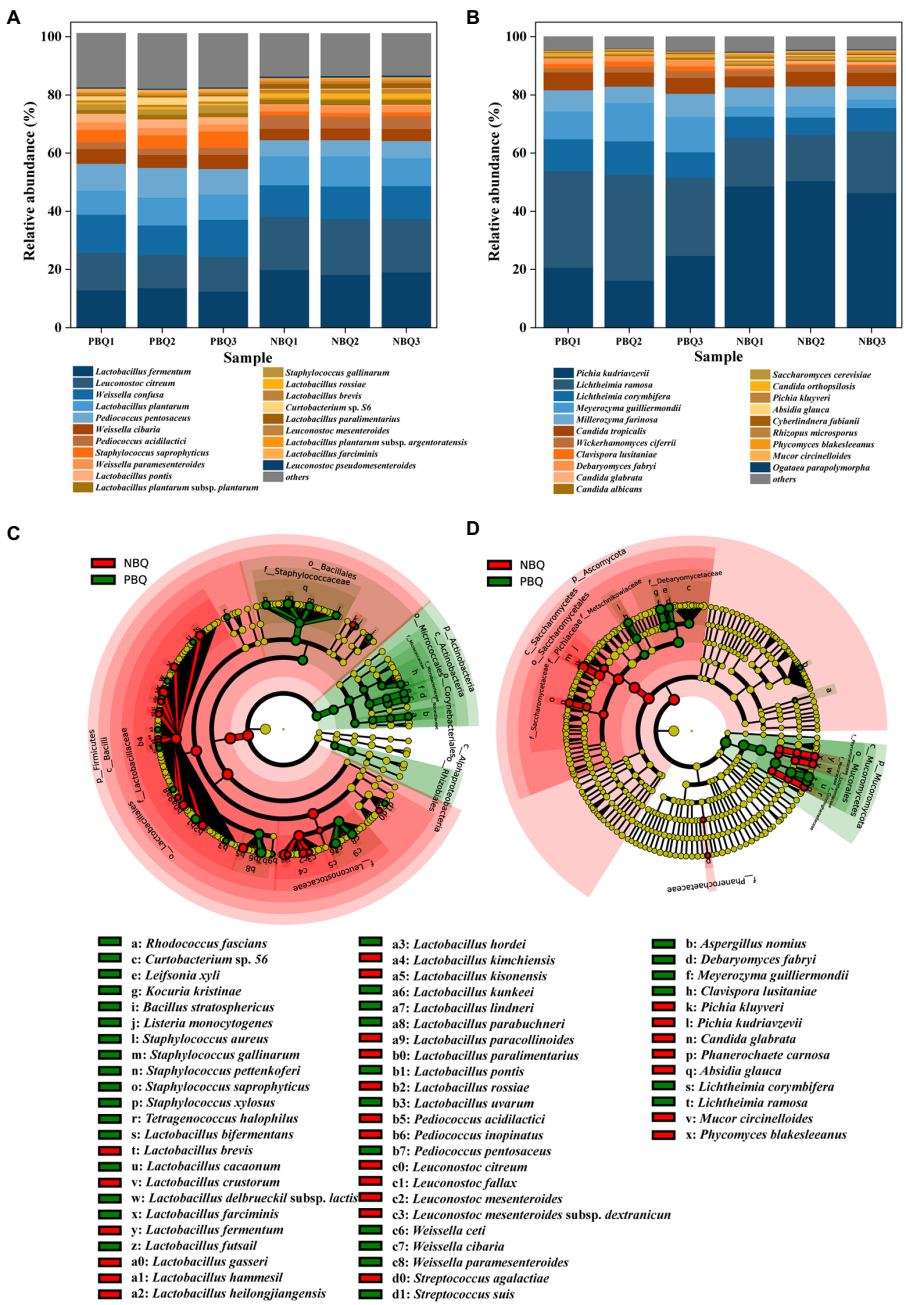
Dominant bacteria **(A)** and fungi **(B)** at species-level; investigation of bacterial **(C)** and fungal **(D)** biomarkers of the two grades of BBQ based on LEfSe.

Taxonomic annotation was performed at the species level and the dominant bacteria and fungi were analyzed. As shown in Figures 2A,B, the species of dominant bacteria and fungi in PBQ and NBQ were similar, but there were differences in relative abundance (RA). Significantly different microorganisms in the two grades of BBQ were analyzed by LEfSe, and the microorganisms with an LDA value greater than 3 were defined as marker microorganisms.

Among the detected bacteria, there were 16 species with RA greater than 1%, mainly belonging to the genera *Lactobacillus*, *Leuconostoc,* and *Weissella*. *Lactobacillus fermentum* was the most dominant bacteria of PBQ and NBQ with RA ranging from 12 to 20%, of which RA in NBQ was higher than that in PBQ. The RA of *Leuconostoc citreum* was between 11 and 20%, which was the second dominant bacteria in the two grades of BBQ, which also had a higher RA in NBQ. According to LEfSe analysis (Figure 2C), *Lactobacillus* and *Leuconostoc* were the marker bacterial genera of NBQ (LDA > 3). Besides the above two dominant bacteria *L. fermentum* and *L. citreum*, *Lactobacillus rossiae*, *Lactobacillus brevis*, and other high-abundance lactic acid bacteria were also biomarkers of NBQ (Figure 2C). The dominant bacterial genera of PBQ were *Weissella* and *Staphylococcus* (LDA > 3), among which *Weissella confusa* was the most abundant, with the RA ranging from 10 to 13%. The RA of *W. confusa* in PBQ was greater than that in NBQ (Figure 2B). Dominant microorganisms including *Weissella cibaria* and *Staphylococcus saprophyticus* were also marker bacteria of PBQ. The differences of bacterial community between PBQ and NBQ were mainly reflected in *Lactobacillus*, *Leuconostoc*, *Weissella* and *Staphylococcus*, which were the acid-producing microorganisms in Baijiu and could provide significant precursors for the formation of esters, and were also conducive to the formation of aromatic alcohols. It suggests that the RA differences of these dominant microorganisms will affect the capacity of aroma production between PBQ and NBQ.

Among the detected fungal species, there were 9 fungi with RA greater than 1%, and the sum of the RA was greater than 90%. *Pichia kudriavzevii* was the most dominant fungi of NBQ and the second dominant fungi of PBQ, accounting for 16% ~ 51%. LEfSe analysis (Figure 2D) indicated that *P. kudriavzevii* was a marker microorganism for NBQ. The higher RA of *P. kudriavzevii* in NBQ might contribute to the stronger ability of aroma production. *Lichtheimia ramosa* was a marker fungus of PBQ, and its RA was much higher than that of NBQ. In addition, dominant fungi such as *Lichtheimia corymbifera*, *Meyerozyma guilliermondii*, *Clavispora lusitaniae,* and *Debaryomyces fabryi* were all the marker microorganisms of PBQ. Higher RA of *L*. *ramosa* and *L*. *corymbifera* in PBQ could be a main reason for the stronger liquefaction and saccharification power.

### The components of volatile compounds in PBQ and NBQ

**Figure 3:**
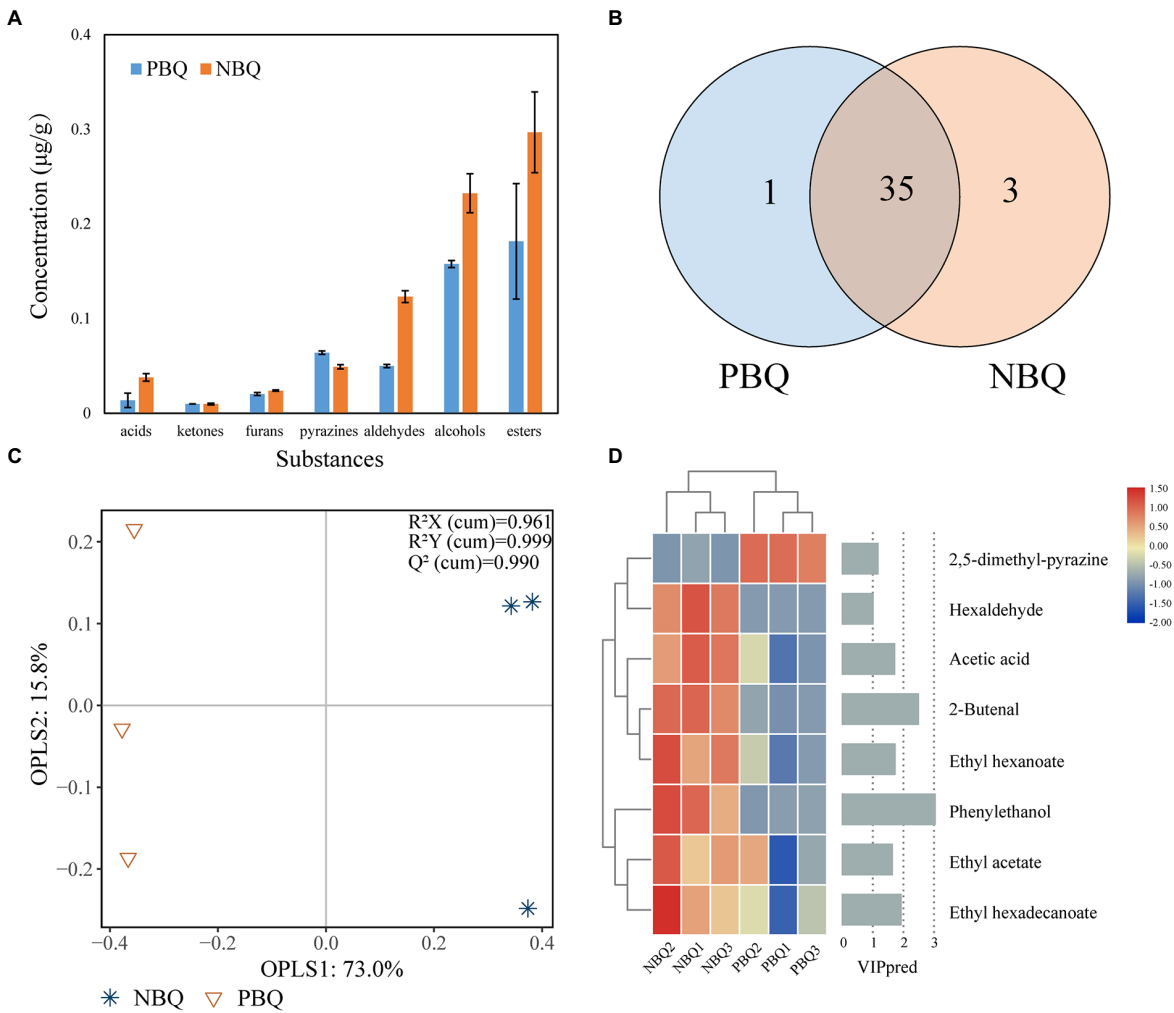
The concentration of 7 categories of substances detected in BBQ **(A)**. Shared and unique volatile substances of PBQ and NBQ **(B)**. Differential analysis of metabolic composition based on OPLS-DA, R2X (*cum*) = 0.961, R2Y (*cum*) = 0.999, Q2 (*cum*) = 0.990 **(C)**. Characteristic substance (VIP > 1, calculated by OPLS-DA) contributing significantly to differences between PBQ and NBQ. The left heatmap represents the content of these 8 characteristic metabolites in the two grades of Daqu, while the right histogram represents the VIP value **(D)**.

A total of 39 volatile compounds were detected in the two types of BBQ, including 3 ketones, 3 furans, 7 pyrazines, 8 aldehydes, 5 alcohols, and 12 esters (Figure 3A). Among them, there are 35 types of substances shared by the two types of BBQ (Figure 3B). Esters, alcohols, and pyrazines are the dominant volatile substances in BBQ (Supplementary Table S2). Except pyrazines, the content of other volatile substances in NBQ was higher than that of PBQ. The unique flavor of PBQ was 2-ethyl-3,5-dimethyl-pyrazine, and the unique flavors of NBQ are (8Z)-1-oxacycloheptadec-8-en-2-one, 3-methylbutanal and hexaldehyde. The OPLS-DA score chart showed that the six samples were grouped into two categories according to the grades, indicating that the composition of metabolites in PBQ and NBQ had significant differences (Figure 3C). It can be seen that a total of 8 metabolites made important contributions to the differences in the metabolic composition of the model (Figure 3D, VIP value>1). The eight substances were selected to draw a heatmap based on the concentration of semi-quantitative substances. The results showed that the content of 2,5-dimethyl-pyrazine in PBQ was significantly higher than that of NBQ, which presents a roasted aroma and was a characteristic flavor substance in PBQ (Gama and Adhikari, 2019). In NBQ, the content of phenylethanol, 2-butenal, hexaldehyde, ethyl acetate, ethyl hexanoate, and ethyl hexadecanoate is higher than that of PBQ, which contributes to the sweet rose-like fragrance (Scognamiglio et al., 2012) and fruity aroma (Xu et al., 2021) and were the were characteristic flavor substances in NBQ.

### The differences in saccharification and liquefaction power between PBQ and NBQ

**Figure 4:**
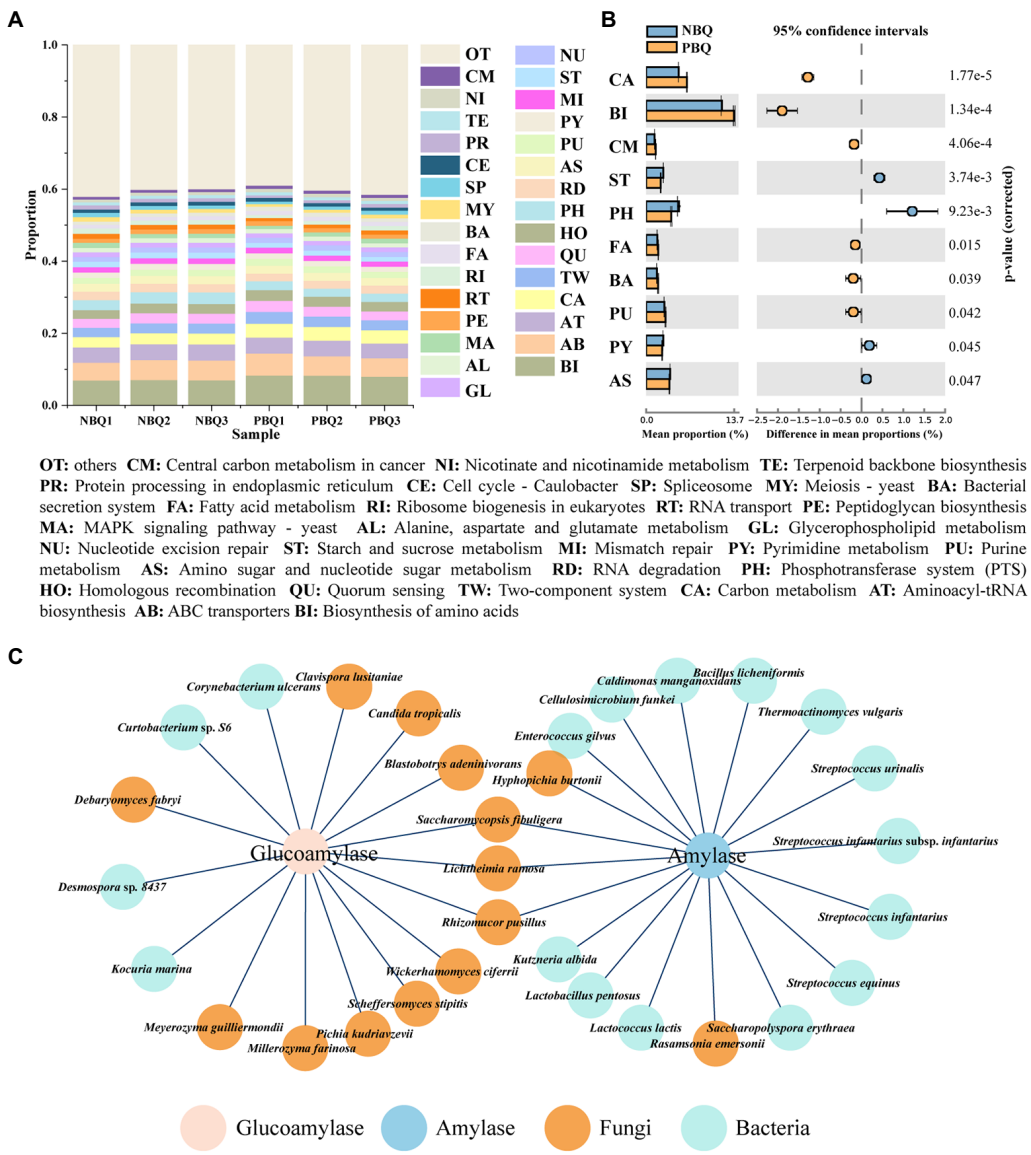
Predominant **(A)** and differential **(B)** metabolic pathways of the two grades of BBQ based on KEGG database; functional microorganisms producing alpha-amylase and glucoamylase based on metagenomic data **(C)**.

The predicted genes were aligned by KEGG database to obtain information on the metabolic functions of the microbial community, which were categorized into three levels of pathways. Biosynthesis of amino acids, ABC transporters, biosynthesis of aminoacyl-tRNA, and carbon metabolism were the most dominant tertiary metabolic pathway in all samples (Figure 4A). There were significant differences between PBQ and NBQ in 10 metabolic pathways (Figure 4B; T-test, *p* < 0.05). Among these pathways, PBQ has advantages in carbon metabolism, biosynthesis of amino acids, fatty acid metabolism, etc. In contrast, NBQ has advantages in starch and sucrose metabolism, pyrimidine metabolism, amino sugar, nucleotide sugar metabolism, etc.

The classification of PBQ and NBQ is divided mainly based on the enzyme activity (saccharification enzyme and amylase), and the activities of enzyme are higher in PBQ. Through the previous results of metaproteomics, it can be seen that the RA of glucoamylase of PBQ and amylase was higher than that of NBQ (Fan et al., 2021). In this study, the microbial sources of the genes encoding glucoamylase (EC 3.2.1.3) and alpha-amylase (EC 3.2.1.1) were investigated based on metagenomic data, and the differences in the ability of starch degradation between PBQ and NBQ were analyzed from the level of microorganisms. It can be seen from Figure 4C that the high-abundance microorganisms producing alpha-amylase in BBQ were *L. ramosa*, *Rasamsonia emersonii*, and *Lactococcus lactis*, and *Lactobacillus pentosus*, while several low-abundance microorganisms also can produce alpha-amylase, including *Rhizomucor pusillus*, *Hyphopichia burtonii*, *Kutzneria albida*, and *Thermoactinomyces vulgaris*. Among these functional microorganisms, there were no significant differences in the RA of enzyme-producing bacteria between PBQ and NBQ. *L. ramosa* was the only significantly differential fungi (Figure 2D, LEfSe, LDA > 3), which was the biomarker of PBQ. The difference of RA between PBQ and NBQ was as high as 14.17%, indicating that the difference in the alpha-amylase-producing ability of the two BBQ was mainly caused by *L. ramosa* that is a common fungus in the manufacturing operation of *Daqu* (Zheng et al., 2015) and in the fermentation process of Baijiu (Huang et al., 2020) and can secrete efficient amylase to degrade wheat (de Oliveira et al., 2016). For glucoamylase, the high-abundance functional microorganisms were *P. kudriavzevii*, *L. ramosa*, *M. guilliermondii*, *Millerozyma Farinosa,* and *Candida Tropicalis*. The RA of functional bacteria was low, including *Curtobacterium* sp. S6, *Desmospora sp*. 8,437, etc. Based on LEfSe analysis, the RA of four fungi (*L. ramosa*, *M. guilliermondii*, *C. lusitaniae*, *D. fabryi*) and one bacterium (*Curtobacterium* sp. S6) in PBQ were significantly higher than those in NBQ, but only the RA for *P. kudriavzevii* was significantly higher in NBQ than PBQ. The results demonstrated that the diversity of functional fungi, which was crucial in the degradation of raw materials, was the primary reason for the differences between NBQ and PBQ.

### The differences in the ability of aroma formation between PBQ and NBQ

**Figure 5:**
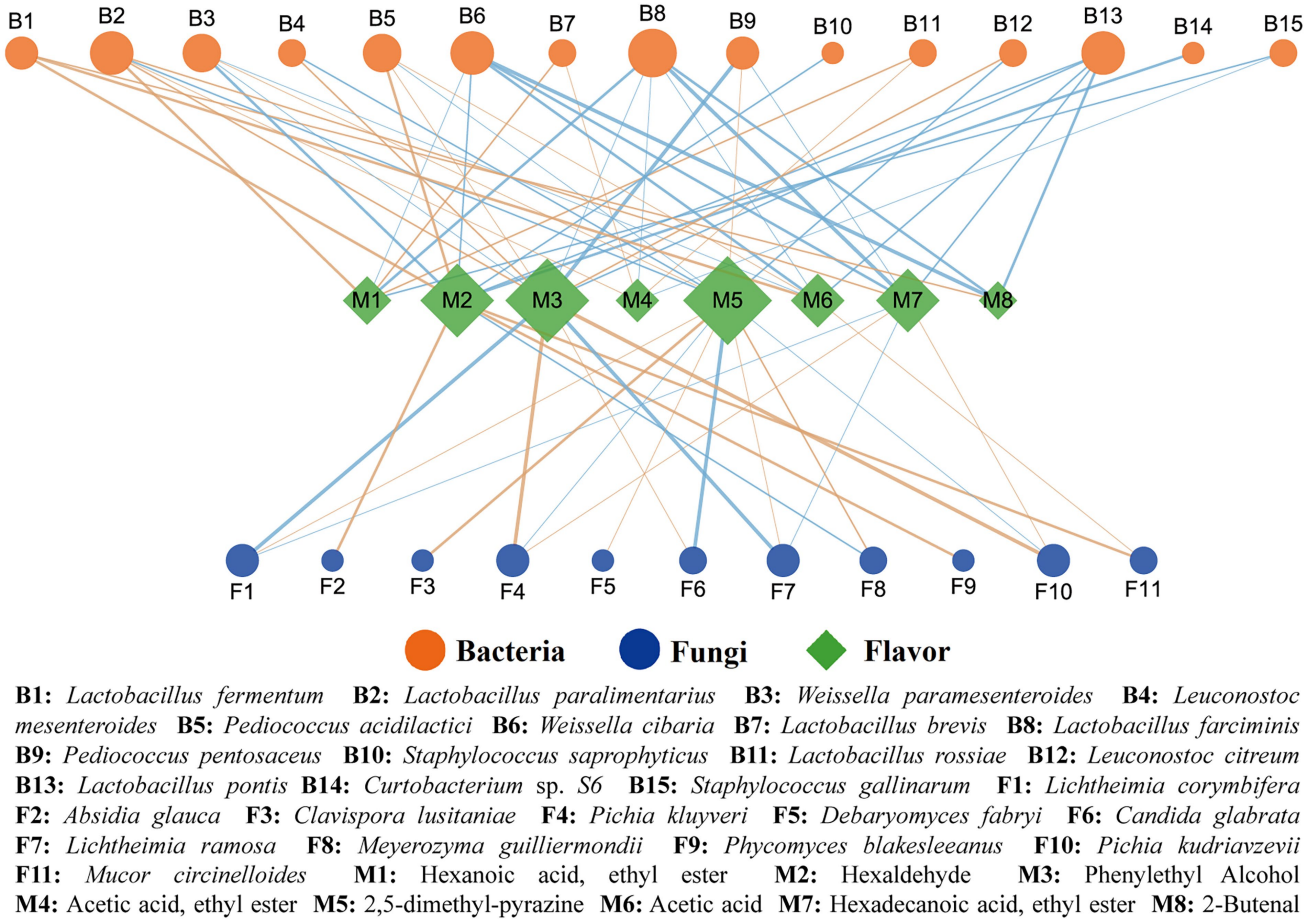
Spearman correlation analysis between dominant differential (LEfSe, LDA > 3) microorganisms and characteristic substance (VIP > 1). The brown edge represents positive correlation, the blue edge represents negative correlation, and the thickness of the edge represents the degree of correlation, the thicker the edge, the stronger the correlation. The size of the node represents the number of objects related to it, the larger the node, the more objects associated with it.

Based on the analysis of Spearman correlation, the microbial factors affecting the metabolic differences between PBQ and NBQ were investigated. The results of visualization (Figure 5) showed that the microorganisms with *p* < 0.05 and |ρ| > 0.6 were significantly correlated with the differential flavors (Figure 3D, VIP > 1). Among all the analyzed microorganisms, bacteria including *Lactobacillus paralimentarius*, *W. cibaria*, *Lactobacillus farciminis*, *Lactobacillus pontis*, and fungi including *L. corymbifera*, *Pichia kluyveri*, *L. ramosa*, *P. kudriavzevii* were related to characteristic metabolites. Ethyl hexanoate and ethyl acetate were only correlated with three *Lactobacillus*: *L. paralimentarius*, *L. brevis*, and *L. rossiae*. Ethyl hexadecanoate was positively correlated with *L. paralimentarius*, *P. kudriavzevii,* and *P. kluyveri*. The results showed that the microorganisms belonged to the genera *Lactobacillus* and *Pichia* played an important role in the formation of esters. The microorganisms mentioned above were biomarkers of NBQ, which were more favorable for the generation of such esters compared with PBQ. Hexaldehyde was significantly positively correlated with *Absidia glauca*, *Phycomyces blakesleanus*, *Mucor circinelloides*, and *Pediococcus acidilactici*, all of which were also marker microorganisms in NBQ. Phenylethanol showed a strong positive correlation with *P. kudriavzevii*, *P. kluyveri*, *L. paralimentarius*, and *P. acidilactici*, of which the RA in NBQ was significantly higher than that in PBQ. It has been reported that phenylethanol is mainly produced through Ehrlich pathway, and the key genes are *AR08*, *AR09*, *AR10*, and *ADH* which correspond to K00838, K05821, K12732, K00001, and K00002 in the KEGG database, respectively. It was found from the metagenomic database that *L. paralimentarius* and *P. acidilactici* had the corresponding functional genes, which proved that they have the potential to generate phenylethanol. Meanwhile, a hypothetical protein detected from *P. kudriavzevii* can function as an ethanol dehydrogenase and it has been proved that *P. kudriavzevii* has the ability to produce phenylethanol (Ogunremi et al., 2020). As the dominant marker microorganism in NBQ, it contributes to the significantly higher content of phenylethanol in NBQ than that in PBQ. The content of 2,5-dimethyl-pyrazine in PBQ was significantly higher than that in NBQ and was strongly positively correlated with biomarkers of PBQ, including *C. lusitaniae*, *D. fabryi*, *L. ramosa*, *M. guilliermondii*, etc. *L. ramosa* could secrete alpha-amylase and glucoamylase, and the other funguses also could secrete glucoamylase, which contributes to the production of glucose through the degradation of starch in the raw materials (wheat and sorghum), thus providing a large number of precursors and energy for the formation of coke flavor substances. Acetic acid, an important precursor of ethyl acetate, was positively correlated with *L. fermentum*, *P. acidilactici*, *Rhizopus microsporus*, *Mucor circineloides*, etc. In addition, 2-butenal was positively correlated with *L. fermentum*.

In general, *Lactobacillus* was a key functional genus in BBQ, and its function was mainly reflected in the generation and accumulation of aromatic alcohols and esters with floral and fruity flavor. The difference in RA of *Lactobacillus* was a remarkable factor causing the differences in aromatic substances between PBQ and NBQ. *P. kudriavzevii*, *L. ramosa,* and *M. guilliermondii* were the dominant functional fungi in BBQ. *P. kudriavzevii* was an important producer of phenylethanol, while *L. ramosa* and *M. guilliermondii* play an important role in the degradation and transformation of starch, which provided an important material basis for the production of pyrogenic substances such as 2,5-dimethyl-pyrazine. Thus, the aroma-producing ability of NBQ was stronger than that of PBQ.

## Conclusion

Due to the long manufacturing period of BBQ, two different grades of BBQ (PBQ and NBQ) are formed and play different roles during the brewing process of Nong-flavor Baijiu. It is necessary to comprehensively analyze the differences between the two types of BBQ in terms of microorganisms, enzymes, and flavor substances. In the previous study, the characterization and comparison of enzymes in two grades of BBQ have been analyzed, and the RA of glycosidase in PBQ was higher than that in NBQ. In this study, the differences between the two grades of BBQ were analyzed at the microbial and metabolic levels. There were certain differences between the two kinds of BBQ in the functional microbiome. The RA of functional microorganisms producing amylase and glucoamylase was significantly different between the two grades of BBQ, making the utilization of raw material much better in PBQ than in NBQ. There were significant differences in the concentration of 8 flavors in the two types of BBQ, including ethyl hexanoate, hexaldehyde, phenylethanol, ethyl acetate, 2,5-dimethyl-pyrazine, acetic acid, ethyl hexadecanoate, and 2-butenal, except for 2,5-dimethyl-pyrazine. The marker microorganisms *Lactobacillus* and *P. kudriavzevii* in NBQ were the key aroma-producing microorganisms. *Lactobacillus* was beneficial to the generation and accumulation of esters and aromatic alcohols, while *P. kudriavzevii* was an important producer of phenylethanol. As a whole, although the structure of the microbial community was similar, the differences in the abundance of functional microorganisms led to the deviations in function. The microbial community of PBQ was inclined to the utilization of raw materials, while the microbial community of NBQ take advantage of forming flavors. In practical production of Baijiu, the mixed use of PBQ and NBQ can complement their functions, which can balance the growth of microorganisms and the formation of flavors. This result can provide theoretical guidance for the following improvement of Baijiu quality.

## Data availability statement

The datasets presented in this study can be found in online repositories. The names of the repository/repositories and accession number(s) can be found in the article/Supplementary material.

## Author contributions

JC, JL, GD, and XZ conceived the idea and the design for this work. QZ carried out the experiments and formal analysis. JZ, ZQ, and DZ provided technical support during the experimental phase. QZ drafted the paper. XZ revised the paper. All authors contributed to the article and approved the submitted version.

## Funding

This article was supported by the National Key Research and Development Program of China (2017YFC1600403), the National First-class Discipline Program of Light Industry Technology and Engineering (LITE2018-08), the National Natural Science Foundation of China (31900067), and the National Key Research and Development Program of China (2019YFA0906400).

## Conflict of interest

QZ, ZQ, JZ, and DZ were employed by the company Wuliangye Yibin Co., Ltd.

The remaining authors declare that the research was conducted in the absence of any commercial or financial relationships that could be construed as a potential conflict of interest.

## Publisher’s note

All claims expressed in this article are solely those of the authors and do not necessarily represent those of their affiliated organizations, or those of the publisher, the editors and the reviewers. Any product that may be evaluated in this article, or claim that may be made by its manufacturer, is not guaranteed or endorsed by the publisher.

## Footnotes

^1^ https://ftp.ncbi.nlm.nih.gov/blast/db/FASTA/

^2^ https://bigd.big.ac.cn/gsa
