# Supplementary material for: Systematic analysis of Baobaoqu fermentation starter for Wuliangye Baijiu by the combination of metagenomics and metabolomics
Source: Front Microbiol. 2022 Dec 1;13:1062547. doi: 10.3389/fmicb.2022.1062547 (PMC9751829; doi:10.3389/fmicb.2022.1062547)
Supplement: Supplementary file 1 [file Data_Sheet_1.docx]

**Systematic analysis of Baobaoqu fermentation starter for *Wuliangye* Baijiu by the combination of metagenomics and metabolomics**

Qingmei Zhang^1,2,6^, Guocheng Du ^1,2,3,4,5^, Jian Chen ^1,2,4,5^, Jianghua Li ^1,2,4,5^, Zongwei Qiao^6^, Jia Zheng^6^, Dong Zhao^6^, Xinrui Zhao ^1,2,4,5*^

^1^ Key Laboratory of Industrial Biotechnology, Ministry of Education, School of Biotechnology, Jiangnan University, 1800 Lihu Road, Wuxi, Jiangsu 214122, China;

^2^ Science Center for Future Foods, Jiangnan University, 1800 Lihu Road, Wuxi, Jiangsu 214122, China;

^3^ Key Laboratory of Carbohydrate Chemistry and Biotechnology, Ministry of Education, Jiangnan University, 1800 Lihu Road, Wuxi, Jiangsu 214122, China;

^4^ Jiangsu Province Engineering Research Center of Food Synthetic Biotechnology, Jiangnan University, 1800 Lihu Road, Wuxi, Jiangsu 214122, China;

^5^ Engineering Research Center of Ministry of Education on Food Synthetic Biotechnology, Jiangnan University, 1800 Lihu Road, Wuxi, Jiangsu 214122, China;

^6^ Wuliangye Yibin Co., Ltd., 150# Minjiang West Road, Cuiping District, Yibin, Sichuan, 644007, China

^*^ Correspondence: zhaoxinrui@jiangnan.edu.cn

Mailing address: School of Biotechnology, Jiangnan University, 1800 Lihu Road, Wuxi, Jiangsu 214122, China.

**Supplementary tables**

**Table S1.** The overall statistics of metagenome for BBQ

| Samples | Clean Number | Base Number | GC Content (%) | %>Q30 |
| --- | --- | --- | --- | --- |
| PBQ1 | 30224350 | 9067305000 | 43.21 | 93.81 |
| PBQ2 | 56926511 | 17077953300 | 44.17 | 93.6 |
| PBQ3 | 46860478 | 14058143400 | 42.94 | 94.12 |
| NBQ1 | 53703634 | 16111090200 | 43.07 | 93.73 |
| NBQ2 | 34064592 | 10219377600 | 42.84 | 93.7 |
| NBQ3 | 48031267 | 14409380100 | 42.99 | 93.94 |

**Table S2.** The concentrations and types of volatile compounds in BBQ

| **Volatile compounds** | **CAS Number** | **Retention time (min)** | **Average concentration**  **(ng/g dry weight)** | | **P-value** |
| --- | --- | --- | --- | --- | --- |
|  |  |  | **PBQ** | **NBQ** |  |
| **Pyrazines** |  |  |  |  |  |
| Methylpyrazine | 109-08-0 | 11.83 | 5.32±0.15 | 4.98±0.15 | 0.0840 |
| 2,6-Dimethyl-pyrazine | 108-50-9 | 13.34 | 16.75±0.95 | 19.26±0.81 | 0.0550 |
| Trimethylpyrazine | 14667-55-1 | 14.90 | 15.68±0.69 | 11.47±1.19 | 0.0161 |
| 2-Ethyl-3,5-dimethyl-pyrazine | 13925-07-0 | 15.93 | 2.13±0.64 | 0±0 | 0.0157 |
| Tetramethylpyrazine | 1124-11-4 | 16.16 | 4.04±0.12 | 4.30±0.38 | 0.3689 |
| 2-Ethyl-6-methyl-pyrazine | 13925-03-6 | 16.36 | 2.85±0.01 | 2.24±0.27 | 0.0401 |
| 2,5-Dimethyl-pyrazine | 123-32-0 | 13.19 | 17.23±0.54 | 6.67±0.50 | 0.0003 |
| **∑(Concentration)** |  |  | 64.00±1.73 | 48.91±2.24 |  |
|  |  |  |  |  |  |
| **Aldehydes** |  |  |  |  |  |
| 3-Methylbutanal | 590-86-3 | 3.90 | 0±0 | 7.05±0.54 | 0.0003 |
| 2-Butenal | 4170-30-3 | 6.29 | 19.17±2.65 | 66.39±4.48 | 0.0008 |
| Hexaldehyde | 66-25-1 | 7.26 | 0±0 | 7.88±1.03 | 0.0012 |
| Nonanal | 124-19-6 | 14.66 | 14.59±0.72 | 21.08±1.96 | 0.0161 |
| Decanal | 112-31-2 | 16.49 | 3.40±0.71 | 3.88±0.59 | 0.4603 |
| Benzaldehyde | 100-52-7 | 16.86 | 10.05±0.41 | 9.82±0.73 | 0.6840 |
| Phenylacetaldehyde | 122-78-1 | 18.55 | 2.42±0.20 | 5.82±0.65 | 0.0042 |
| 2-Phenylcrotonaldehyde | 4411-89-6 | 22.00 | 0.21±0.06 | 1.28±0.33 | 0.0161 |
| **∑(Concentration)** |  |  | 49.85±1.8 | 123.17±6.25 |  |
|  |  |  |  |  |  |
| **Esters** |  |  |  |  |  |
| Ethyl acetate | 141-78-6 | 3.51 | 58.28±24.46 | 88.24±10.87 | 0.1676 |
| Ethyl hexanoate | 123-66-0 | 11.11 | 29.46±6.2 | 53.99±5.13 | 0.0161 |
| Ethyl heptanoate | 106-30-9 | 13.45 | 9.04±1.67 | 10.94±0.99 | 0.2068 |
| Ethyl octanoate | 106-32-1 | 15.43 | 16.15±4.90 | 20.72±2.64 | 0.2779 |
| Ethyl nonanoate | 123-29-5 | 17.05 | 6.63±0.81 | 6.43±0.91 | 0.8137 |

**Table S2.** The concentrations and types of volatile compounds in BBQ (continued)

| **Volatile compounds** | **CAS Number** | **Retention time (min)** | **Average concentration**  **(ng/g dry weight)** | | **P-value** |
| --- | --- | --- | --- | --- | --- |
|  |  |  | **PBQ** | **NBQ** |  |
| Ethyl decanoate | 110-38-3 | 18.50 | 1.63±0.47 | 3.28±0.89 | 0.0834 |
| Ethyl dodecanoate | 106-33-2 | 20.97 | 1.46±0.42 | 2.60±0.54 | 0.0834 |
| Ethyl tetradecanoate | 124-06-1 | 23.13 | 4.13±1.17 | 8.27±1.88 | 0.0658 |
| Ethyl pentadecanoate | 41114-00-5 | 24.13 | 0.72±0.19 | 1.20±0.28 | 0.1039 |
| Ethyl hexadecanoate | 628-97-7 | 25.08 | 40.32±15.62 | 75.07±15.5 | 0.0848 |
| Ethyl (9E)-9-octadecenoate | 6114-18-7 | 27.19 | 5.03±2.53 | 10.84±2.28 | 0.0816 |
| 9,12-Octadecadienoic acid, methyl ester | 2566-97-4 | 27.73 | 8.78±3.93 | 15.28±3.02 | 0.1191 |
| **∑(Concentration)** |  |  | 181.64±60.89 | 296.86±42.67 |  |
|  |  |  |  |  |  |
| **Alcohols** |  |  |  |  |  |
| 2-Ethylhexanol | 104-76-7 | 8.95 | 43.06±0.01 | 44.92±0.02 | 0.9055 |
| 2-Methyl-1-butanol | 137-32-6 | 10.51 | 27.26±0 | 34.29±0 | 0.1191 |
| 1-Hexanol | 111-27-3 | 13.90 | 12.20±0 | 7.38±0 | 0.0003 |
| Phenylmethanol | 100-51-6 | 21.38 | 7.82±0 | 7.15±0 | 0.3599 |
| Phenylethanol | 60-12-8 | 21.78 | 67.26±0 | 138.69±0.02 | 0.0079 |
| **∑(Concentration)** |  |  | 157.59±0 | 232.44±0.02 |  |
|  |  |  |  |  |  |
| **Ketones** |  |  |  |  |  |
| 2-Nonanone | 821-55-6 | 14.58 | 6.93±0.17 | 4.11±0.50 | 0.0038 |
| 2-Octanone | 111-13-7 | 17.97 | 2.80±0.16 | 3.02±0.29 | 0.3655 |
| (8Z)-1-oxacycloheptadec-8-en-2-one | 123-69-3 | 23.53 | 0±0 | 2.63±0.36 | 0.0012 |
| **∑(Concentration)** |  |  | 9.73±0.12 | 9.75±0.89 |  |
|  |  |  |  |  |  |
| **Furans** |  |  |  |  |  |
| 2-Pentylfuran | 3777-69-3 | 11.02 | 13.87±1.21 | 15.45±0.84 | 0.1772 |
| 2-Furanmethanol | 98-00-0 | 18.76 | 3.72±0.23 | 4.23±0.24 | 0.0882 |
| 2-Furancarboxaldehyde | 98-01-1 | 15.86 | 2.69±0.13 | 4.20±0.10 | 0.0008 |
| **∑(Concentration)** |  |  | 20.28±1.55 | 23.88±0.62 |  |
|  |  |  |  |  |  |
| **Acid** |  |  |  |  |  |
| Acetic acid | 64-19-7 | 15.70 | 0.01±0.01 | 0.04±0.01 | 0.0191 |
| **∑(Concentration)** |  |  | 0.01±0.01 | 0.04±0.01 |  |
|  |  |  |  |  |  |
| **∑(Type) in total** |  |  | 36 | 38 |  |
